# Estimating variance components in population scale family trees

**DOI:** 10.1371/journal.pgen.1008124

**Published:** 2019-05-09

**Authors:** Tal Shor, Iris Kalka, Dan Geiger, Yaniv Erlich, Omer Weissbrod

**Affiliations:** 1 Computer Science Department, Technion—Israel Institute of Technology, Haifa, Israel; 2 MyHeritage Ltd., Or Yehuda, Israel; 3 Department of Computer Science and Applied Mathematics, Weizmann Institute of Science, Rehovot, Israel; 4 Department of Molecular Cell Biology, Weizmann Institute of Science, Rehovot, Israel; 5 The New York Genome Center, New York, NY, United States of America; 6 Department of Computer Science, Fu School of Engineering, Columbia University, NY, United States of America; 7 Department of Epidemiology, Harvard T.H. Chan School of Public Health, Boston, MA, United States of America; The University of Queensland, AUSTRALIA

## Abstract

The rapid digitization of genealogical and medical records enables the assembly of extremely large pedigree records spanning millions of individuals and trillions of pairs of relatives. Such pedigrees provide the opportunity to investigate the sociological and epidemiological history of human populations in scales much larger than previously possible. Linear mixed models (LMMs) are routinely used to analyze extremely large animal and plant pedigrees for the purposes of selective breeding. However, LMMs have not been previously applied to analyze population-scale human family trees. Here, we present **S**parse **C**holesky factor**I**zation LMM (Sci-LMM), a modeling framework for studying population-scale family trees that combines techniques from the animal and plant breeding literature and from human genetics literature. The proposed framework can construct a matrix of relationships between trillions of pairs of individuals and fit the corresponding LMM in several hours. We demonstrate the capabilities of Sci-LMM via simulation studies and by estimating the heritability of longevity and of reproductive fitness (quantified via number of children) in a large pedigree spanning millions of individuals and over five centuries of human history. Sci-LMM provides a unified framework for investigating the epidemiological history of human populations via genealogical records.

## Author summary

The advent of online genealogy services allows the assembly of population-scale family trees, spanning millions of individuals and centuries of human history. Such datasets enable answering genetic epidemiology questions on unprecedented scales. Here we present Sci-LMM, a pedigree analysis framework that combines techniques from animal and plant breeding research and from human genetics research for large-scale pedigree analysis. We apply Sci-LMM to analyze population-scale human genealogical records, spanning trillions of relationships. We have made both Sci-LMM and an anonymized dataset of millions of individuals freely available to download, making the analysis of population-scale human family trees widely accessible to the research community. Together, these resources allow researchers to investigate genetic and epidemiological questions on an unprecedented scale.

## Introduction

Genealogical records can reflect social and cultural structures, and record the flow of genetic material throughout history. In recent years, very large pedigree records have come into existence, owing to collaborative digitization of large genealogical records [1,2] and to digitization of large cohorts collected by healthcare providers, spanning up to millions of individuals [3–7]. Such population-scale pedigrees allow investigating the sociological and epidemiological history of human populations on a scale that is orders of magnitude larger than existing studies.

Traditional human pedigree studies collect a large number of independent families which are analyzed separately and then meta-analyzed. However, this approach is not suitable for population-scale pedigrees, because such pedigrees cannot be decomposed into mutually exclusive families [1]. Hence, the analysis of such pedigrees requires modeling complex covariance structures between trillions of pairs of individuals.

Pedigree studies often employ LMMs to decompose the phenotypic variation among individuals into variance components such as genetic effects and shared environment [8]. LMMs have been the statistical backbone of animal and plant breeding programs for almost six decades [9], and have been continuously developed over the years [10–22]. LMMs are routinely used nowadays to analyze pedigrees of millions of animals and plants [13,23], hundreds of thousands of which are often genotyped (e.g. [22,24,25]).

LMMs and their extensions have recently gained considerable popularity in human genetics studies for the purposes of estimating heritability [26–32] and genetic correlation [33–37], predicting phenotypes [38–41] and modeling sample relatedness [42–46]. Unlike classical animal and plant studies, human studies typically do not include pedigree data, but instead measure genetic relatedness via dense genotyping of single nucleotide polymorphisms (SNPs).

In recent years, animal and human studies have been using different techniques to scale LMMs to datasets with millions of individuals. Animal studies typically fit large-scale LMMs via restricted maximum likelihood (REML) [12], by exploiting the sparsity of pedigree data. Specifically, a pair of individuals with no known common ancestor can be regarded as having no genetic similarity. Consequently, these pairs induce a zero entry in the genetic similarity matrix. Such sparse matrices can be stored and analyzed efficiently with suitable numerical techniques [21,47,48].

Human genotyping studies do not give rise to sparse data structures. Instead, human studies have managed to scale LMMs to large datasets via two approaches. The first approach applies REML, either via supercomputers with thousands of CPUs and terabytes of memory [46], or by approximating the restricted likelihood gradient via Monte-Carlo techniques [28]. However, the latter technique is only suitable for specific types of covariance matrices whose decomposition is known beforehand.

The second approach to scale LMMs uses the method of moments rather than REML, by solving a set of second moment matching equations [49–53]. Such approaches have become increasingly popular recently [30–35,54–60] owing to their computational tractability and their compatibility with privacy-preserving summary statistics [61]. Although moment estimators are less statistically efficient compared to REML estimators, they have several advantages: the loss of efficiency has been found to often be small [56]; they are more robust to modeling violation because they make fewer distributional assumptions; and they are more flexible, which enables applying techniques to limit confounding factors such as assortative mating (Methods). Moment estimators have also recently been explored in animal breeding studies [62–64] and were found to be faster than REML while providing similar accuracy, but they have not been widely adopted in animal studies to date.

Here we present Sci-LMM, a statistical framework for analyzing population-size pedigrees that combines techniques from animal and human genetic studies. Sci-LMM uses sparse data structures as is common in animal studies, and supports both moment and REML estimators. The moment estimator is based on a common technique called Haseman-Elston (HE) regression [65,66] (Methods). Sci-LMM scales HE regression to population-sized pedigrees via sparse matrix tools [67]. The REML estimator combines a direct sparse REML solver [47] with Monte-Carlo gradient approximation [68]. Importantly, existing packages for pedigree-based REML [69–73] cannot handle the analyses performed in this paper because they require the inverse of the epistatic interactions matrix [47,74,75], which is extremely difficult to compute in large pedigrees [76]. Hence, Sci-LMM provides a comprehensive solution for LMM-based pedigree analysis.

To demonstrate the capabilities of Sci-LMM, we carry out an extensive analysis of simulated data with millions of individuals, which we complete within a few hours. We additionally estimate the heritability of longevity and of reproductive fitness (quantified via number of children), using a large cohort spanning millions of genealogical records and several centuries of human history. We estimate that both traits have a substantial heritable component, with an estimated 22.1% heritability for longevity and 34.4% for reproductive fitness. Sci-LMM enables analysis of large pedigree records that was not previously possible.

## Material and methods

### Linear mixed models

Consider a sample of *n* individuals with observed phenotypes *y*_1_,…,*y*_*n*_, and covariates vectors ***C***_1_,…,***C***_*n*_, and consider a set of *n*×*n* covariance matrices ***M***^1^,…,***M***^*d*^, where $M_{i,j}^{k}$ encodes the covariance between the phenotypes of individuals *i* and *j* according to the *k*^th^ covariance structure, up to a scaling constant. We assume that the vector ***y*** = [*y*_1_,…,*y*_*n*_]^*T*^ follows a multivariate normal distribution:

y∼N(Cβ;Σ) (1)

$$\Sigma =G+\sigma_{e}^{2}I$$ (2)

$$G=\sum_{k=1}^{d}\sigma_{k}^{2}M^{k}.$$ (3)

Here, ***C*** = [***C***_1_,…,***C***_*n*_]^*T*^ is an *n*×*c* matrix of covariates (including an intercept), ***β*** is a *c*×1 vector of fixed effects, **Σ** is the covariance matrix of the vector ***y***, $\sigma_{k}^{2}$ is the k^th^ variance component, and ***I*** is the identity matrix. The parameters to estimate are the fixed effects ***β*** and the variance components $\sigma_{1}^{2},\ldots ,\sigma_{d}^{2},\sigma_{e}^{2}$. The Sci-LMM software can currently compute an identity by descent (IBD) matrix, an epistatic covariance matrix and a dominance matrix, as described below.

The restricted log-likelihood $l_{R}(\beta ,\sigma_{1}^{2},\ldots ,\sigma_{d}^{2},\sigma_{e}^{2})$ is given by [77]:

$$l_{R}(\beta ,\sigma_{1}^{2},\ldots ,\sigma_{d}^{2},\sigma_{e}^{2})=l(\beta ,\sigma_{1}^{2},\ldots ,\sigma_{d}^{2},\sigma_{e}^{2})+\frac{c}{2}\log\left(2\pi \right)+\frac{1}{2}log|C^{T}C|-\frac{1}{2}log|C^{T}\Sigma^{-1}C|$$ (4)

$$l(\beta ,\sigma_{1}^{2},\ldots ,\sigma_{d}^{2},\sigma_{e}^{2})=-\frac{1}{2}(y-C\beta )\Sigma^{-1}(y-C\beta )-\frac{1}{2}log|\Sigma |-\frac{n}{2}\log\left(2\pi \right),$$ (5)

where $l(\beta ,\sigma_{1}^{2},\ldots ,\sigma_{d}^{2},\sigma_{e}^{2})$ is the non-restricted likelihood and *c* is the number of covariates.

An alternative form of Eq 4 often used in animal breeding literature is:

$$l_{R}(\beta ,\sigma_{1}^{2},\ldots ,\sigma_{d}^{2},\sigma_{e}^{2})=-\frac{1}{2}(\log|B|+\log|\Sigma |+y^{T}Py),$$ (6)

where $B=\left[\begin{array}{cc} \sigma_{e}^{-2}C^{T}C & {\sigma_{e}^{-2}C}^{T}\\ \sigma_{e}^{-2}C & \sigma_{e}^{-2}I+G^{-1} \end{array}\right],\;P=\sigma_{e}^{-2}I-\sigma_{e}^{-4}WB^{-1}W^{T},\;W=[C\;I]$ and we ignored additive constants. This form is particularly convenient when the inverse of each of the matrices ***M***^1^,…,***M***^*d*^ is known, as it can be solved efficiently using mixed model equations via Gaussian elimination, without having to directly invert or factorize the matrix **Σ** [47,72]. This makes it particularly convenient to use this form in the presence of only an additive IBD matrix, because the inverse of this matrix is sparse and can be computed analytically [78,79].

### Restricted maximum likelihood (REML) estimation

REML estimation consists of finding the parameters $\beta ,\sigma_{1}^{2},\ldots ,\sigma_{d}^{2},\sigma_{e}^{2}$ that maximize Eq 4. When the inverse of each of the matrices ***M***^1^,…,***M***^*d*^ is known, the REML can be found efficiently by using Eq 6, using the so-called mixed model equations method [47,72]. Here we describe a direct solution that can be applied when the inverse of ***M***^1^,…,***M***^*d*^ is unknown.

Our solution combines several ideas: (1) we maximize Eq 4 directly, rather than the equivalent form of Eq 6; (2) instead of directly inverting **Σ**, we compute its Cholesky factorization **Σ** = ***LL***^*T*^ via sparse matrix routines; (3) any product of the form **Σ**^−1^***v*** for some vector ***v*** is computed using L and two triangular solvers (forward and backward substitution); and (4) the gradient of Eq 4 is approximated using Monte Carlo techniques. We now describe our REML approach in detail.

We first describe a solution to the unrestricted log-likelihood (Eq 5) and then extend the solution to the restricted log-likelihood (Eq 4). To compute Eq 5 we need to compute the terms **Σ**^−1^(**y**−**Cβ**) and log|**Σ**|. The first term can be computed exactly via either conjugate gradient iterations or by explicitly computing the Cholesky factorization of **Σ** and then applying forward and back substitution. The second term can be computed via the Cholesky factorization of **Σ**. The Cholesky factorization can be computed efficiently via the CHOLMOD routines [80]. It remains to find the maximum likelihood estimates of the model parameters.

To find the MLE of $\hat{\beta }$ we note that given **Σ**, $\hat{\beta }$ can be computed analytically by deriving Eq 5 with respect to **β** as follows:

$$\frac{\partial l(\beta ,\sigma_{1}^{2},\ldots ,\sigma_{d}^{2},\sigma_{e}^{2})}{\partial \beta }=-\frac{1}{2}(y-C\beta {)}^{T}\Sigma^{-1}C$$ (7)

By setting the transpose of the gradient to 0, we obtain the MLE:

$$\hat{\beta }={\left(C^{T}\Sigma^{-1}C\right)}^{-1}C^{T}\Sigma^{-1}y.$$ (8)

The MLEs of the variance components ${\hat{\sigma }}_{1}^{2},\ldots ,{\hat{\sigma }}_{d}^{2},{\hat{\sigma }}_{e}^{2}$ are estimated via an optimization procedure, which requires computing the gradient of Eq 5. The partial derivative with respect to each variance component $\sigma_{k}^{2}$ is given by:

$$\frac{\partial l(\beta ,\sigma_{1}^{2},\ldots ,\sigma_{d}^{2},\sigma_{e}^{2})}{\partial \sigma_{k}^{2}}=-\frac{1}{2}y^{T}\Sigma^{-1}M^{k}\Sigma^{-1}y-\frac{1}{2}Tr[\Sigma^{-1}M^{k}].$$ (9)

The first term on the right-hand side of Eq 9 can be computed efficiently given the Cholesky factorization of **Σ**. Unfortunately, the second term cannot be solved efficiently via the above technique because it requires solving *n* different linear equations, where *n* can be in the millions. Instead, we use the approximation technique used in [28,68,81]. We first rewrite this term as an expectation (ignoring the scaling factor) as follows:

$$\begin{array}{l} Tr[\Sigma^{-1}M^{k}]=Tr[\Sigma^{-1}M^{k}\Sigma^{-1}\Sigma ]\\ \;=Tr\left[\Sigma^{-1}M^{k}\Sigma^{-1}E[y\prime y\prime^{T}]\right]\\ \;=E\left[Tr[\Sigma^{-1}M^{k}\Sigma^{-1}y\prime y\prime^{T}]\right]\\ \;=E\left[Tr[y\prime^{T}\Sigma^{-1}M^{k}\Sigma^{-1}y\prime ]\right]\\ \;=E[y\prime^{T}\Sigma^{-1}M^{k}\Sigma^{-1}y\prime ], \end{array}$$ (10)

where y′∼N(0,Σ) and we used the fact that the trace of a scalar is equal to the scalar. We therefore approximate Eq 10 by sampling a small number of **y**′ vectors to approximate the expectation. These vectors can be sampled efficiently given the Cholesky factorization **Σ** = **LL**^*T*^ by sampling a vector $y_{\Sigma }∼N(0,I)$ and then using the fact that $Ly_{\Sigma }∼N(0,\Sigma )$. The Cholesky factorization can be computed efficiently via the CHOLMOD routines. We found that 100 vectors often yields a very good approximation at a modest computational cost.

We note that [28] proposes an alternative estimation method by completely foregoing the likelihood computation, and instead only trying to minimize the squared gradient elements. However, we found that in sparse settings, this solution often converges into local maxima at the edge of the parameter space (where many variance components are equal to zero) rather than the true maximum likelihood estimate.

We now extend the solution to handle restricted maximum likelihood (Eq 4). Clearly, the restricted maximum likelihood estimate of **β** is the same as the MLE. The derivative of the restricted log likelihood with respect to each variance component $\sigma_{k}^{2}$ is given by:

$$\frac{\partial l_{R}(\beta ,\sigma_{1}^{2},\ldots ,\sigma_{d}^{2},\sigma_{e}^{2})}{\sigma_{k}^{2}}=\frac{\partial l(\beta ,\sigma_{1}^{2},\ldots ,\sigma_{d}^{2},\sigma_{e}^{2})}{\sigma_{k}^{2}}+\frac{1}{2}Tr[{\left(C^{T}\Sigma^{-1}C\right)}^{-1}C^{T}\Sigma^{-1}M^{k}\Sigma^{-1}C].$$ (11)

The term **Σ**^−1^**C** can be computed by solving *c* different linear equations, which can be performed efficiently given the Cholesky factorization of **Σ**. All the other terms can be computed efficiently, assuming that *c* is small compared to *n*.

The standard errors of $\sigma_{1}^{2},\ldots ,\sigma_{d}^{2}$ can be approximated via the average information REML (AI-REML) procedure [82], which consists of approximating each entry of the Hessian of the restricted log likelihood as follows:

$$\frac{\partial l(\beta ,\sigma_{1}^{2},\ldots ,\sigma_{d}^{2},\sigma_{e}^{2})}{\sigma_{k}^{2}\sigma_{l}^{2}}\approx -\frac{1}{2}y^{T}PM^{k}PM^{l}Py,$$ (12)

where **P** = **Σ**^−1^− **Σ**^−1^**C**(**C**^*T*^**Σ**^−1^**C**)^−1^**C**^*T*^**Σ**^−1^. Afterwards we approximate the standard errors via the square roots of the diagonal entries of the inverse of the negative Hessian. Following [28], we multiply these entries by $\left(1+\frac{1}{100}\right)$ to account for sampling variance introduced by the 100 ***y***′ vectors sampled in the Monte-Carlo approximation.

### Implementation details

We implemented our REML solver in Python, using an L-BFGS-B algorithm [83] as implemented in the SciPy package [67]. To prevent the parameters from inducing a non positive-definite matrix, We enforced non-negative parameters by using a log-transformation, which transforms the problem into an unconstrained optimization problem.

### Haseman-Elston regression

HE regression estimates variance components via the method of moments, by finding the set of variance components $\sigma_{1}^{2},\ldots ,\sigma_{d}^{2},\sigma_{e}^{2}$ that minimize the expression:

$$\sum_{i,j}{\left(cov(y_{i}-C_{i}\beta ,y_{j}-C_{j}\beta )-\Sigma_{ij}\right)}^{2}.$$ (13)

Typically, the fixed effects ***β*** are first estimated without considering the covariance matrices, by solving the multivariate linear regression problem $y=C\beta +\epsilon ,\epsilon ∼N(0,\sigma_{e}^{2}I)$, where ***I*** is the identity matrix. This solution yields a consistent estimator under mild regularity conditions [84]. Afterwards we plug the fixed effect estimate $\hat{\beta }$ into Eq 13 and estimate the variance component estimates ${\hat{\sigma }}_{1}^{2},\ldots ,{\hat{\sigma }}_{d}^{2}$ as follows:

$${\left[{\hat{\sigma }}_{1}^{2},\ldots ,{\hat{\sigma }}_{d}^{2}\right]}^{T}={\left({\left[V^{1},\ldots ,V^{d}\right]}^{T}[V^{1},\ldots ,V^{d}]\right)}^{-1}{\left[V^{1},\ldots ,V^{d}\right]}^{T}Y,$$ (14)

where **V**^*k*^ is a vector representation enumerating the elements $M_{ij}^{k}$ for all pairs of distinct individuals *i*,*j*, and **Y** is a vector representation of the corresponding elements $\left(y_{i}-C_{i}\hat{\beta })(y_{j}-C_{j}\hat{\beta }\right)$. Each element *q*,*r* of the *d*×*d* matrix ([**V**^1^,…,**V**^*d*^]^*T*^[**V**^1^…,**V**^*d*^]) can be computed via an element-wise multiplication of the upper-diagonal elements of the matrices **M**^*q*^, **M**^*r*^, which can be performed efficiently via sparse matrix routines. The vector [**V**^1^,…,**V**^*d*^]^*T*^**Y** can also be computed efficiently in a similar manner.

By following the notation of [56] and denoting **q**≜[**V**^1^,…,**V**^*d*^]^*T*^**Y**, **S** = [**V**^1^,…,**V**^*d*^]^*T*^[**V**^1^,…,**V**^*d*^], we have:

$${\left[{\hat{\sigma }}_{1}^{2},\ldots ,{\hat{\sigma }}_{d}^{2}\right]}^{T}=S^{-1}q.$$ (15)

By applying a few matrix manipulations, we can compute **q** and **S** efficiently as follows:

$$q_{k}=y^{T}M^{k}y-\sum_{i}M_{ii}^{k}y_{i}^{2}=y^{T}(M^{k}-I)y$$ (16)

$$S_{kl}=\sum_{i,j}M_{ij}^{k}M_{ij}^{l}-\sum_{i}M_{ii}^{k}M_{ii}^{l},$$ (17)

where we used the assumption $M_{ii}^{k}=1$. Both these quantities can be computed explicitly via sparse matrix routines.

The sampling variance of the estimators is given by **S**^−1^var(**q**)**S**^−1^, where var(**q**) is given by:

$$var{\left(q\right)}_{kl}=2tr\left(\hat{\Sigma }(M^{k}-I)\hat{\Sigma }(M^{l}-I)\right).$$ (18)

This quantity can be computed in two ways:

1. Exactly, via: $tr\left(\hat{\Sigma }(M^{k}-I)\hat{\Sigma }(M^{l}-I)\right)=\sum_{ij}{\left[\hat{\Sigma }(M^{k}-I)\right]}_{ij}{\left[\hat{\Sigma }(M^{l}-I)\right]}_{ij}$
2. Approximately, via: $tr\left(\hat{\Sigma }(M^{k}-I)\hat{\Sigma }(M^{l}-I)\right)=E_{y\prime }[y\prime^{T}(M^{k}-I)\hat{\Sigma }(M^{l}-I)y\prime ],$

where **y**′ is sampled from N(0,I), using a derivation similar to the one in Eq 10.

The approximate approach uses Monte-Carlo approximations, by randomly sampling **y**′ vectors and approximating the right hand side. It can be substantially faster than the exact approach (because it circumvents expensive matrix-matrix multiplications) and obtain excellent accuracy. The Sci-LMM software uses the approximate approach by sampling ~100 random **y**′ vectors.

HE regression provides a simple technique for excluding specific pairs of individuals (e.g. spouses) from the analysis without excluding the individuals themselves. This can be useful when trying to limit confounding due to factors such as assortative mating. Excluded pairs can be omitted by zeroing the covariance matrix entries of corresponding pairs. Importantly, this technique cannot be used in REML, because the resulting covariance matrices may not be positive definite. We note that another potential approach to capture environmental risk factors is including shared effects with a suitable incidence matrix [10], but this approach requires additional assumptions and has not been used here.

### Factors affecting estimation accuracy

HE regression is a convenient theoretical framework to analyze the factors affecting estimation accuracy. HE regression can be considered as a special form of linear regression, where off-diagonal entries of covariance matrices serve as explanatory variables. Hence, good accuracy is obtained when measured and unmeasured explanatory variables are uncorrelated with other explanatory variables (Eq 18).

Specifically, obtaining accurate estimates requires (1) that the off-diagonal entries of the LMM covariance matrices are uncorrelated with each other; and (2) that they are uncorrelated with covariance due to unmeasured environmental factors. While the first requirement can be easily tested, the second one requires making strong assumptions about the structure of environmental covariance. For example, if latent environmental factors are shared between spouses but not between parents and children, we may wish to exclude spouses from the analysis. Unfortunately, we not know the structure of environmental covariance for the traits studied in this work, and we leave its investigation for future work.

### Identity by descent matrix

The IBD kinship coefficient of two individuals, denoted as *a*_*ij*_, is the probability that a randomly selected allele in an autosomal locus was originated from the same chromosome of a shared ancestor between individuals *i* and *j* [85,86], and is given by:

$$a_{i,j}=\left\{ \begin{array}{l} \;1+f_{i},\;i=j\\ r_{ij}\sqrt{a_{ii}a_{jj}},\;i\neq j \end{array}\right.$$

Here, *f*_*i*_ is the inbreeding coefficient, defined as half of the IBD coefficient of the parents of individual *i* [85], and *r*_*ij*_ is the coefficient of relationships, defined as:

$$r_{ij}=\frac{\sum_{path}\frac{1+f_{A}}{2^{|path|+1}}}{\sqrt{\left(1+f_{i})(1+f_{j}\right)}}$$

The quantities in the above equation are defined as follows: *A* is a least common ancestor of individual *i* and *j* in the pedigree graph (a graph where every node is an individual connected to her parents and children); the summation is performed over every path connecting individuals *i*,*j* in the pedigree graph, culminating at some ancestor A, such that the path does not contain the same individual twice; and |path| is the path length.

**Fig 1:**
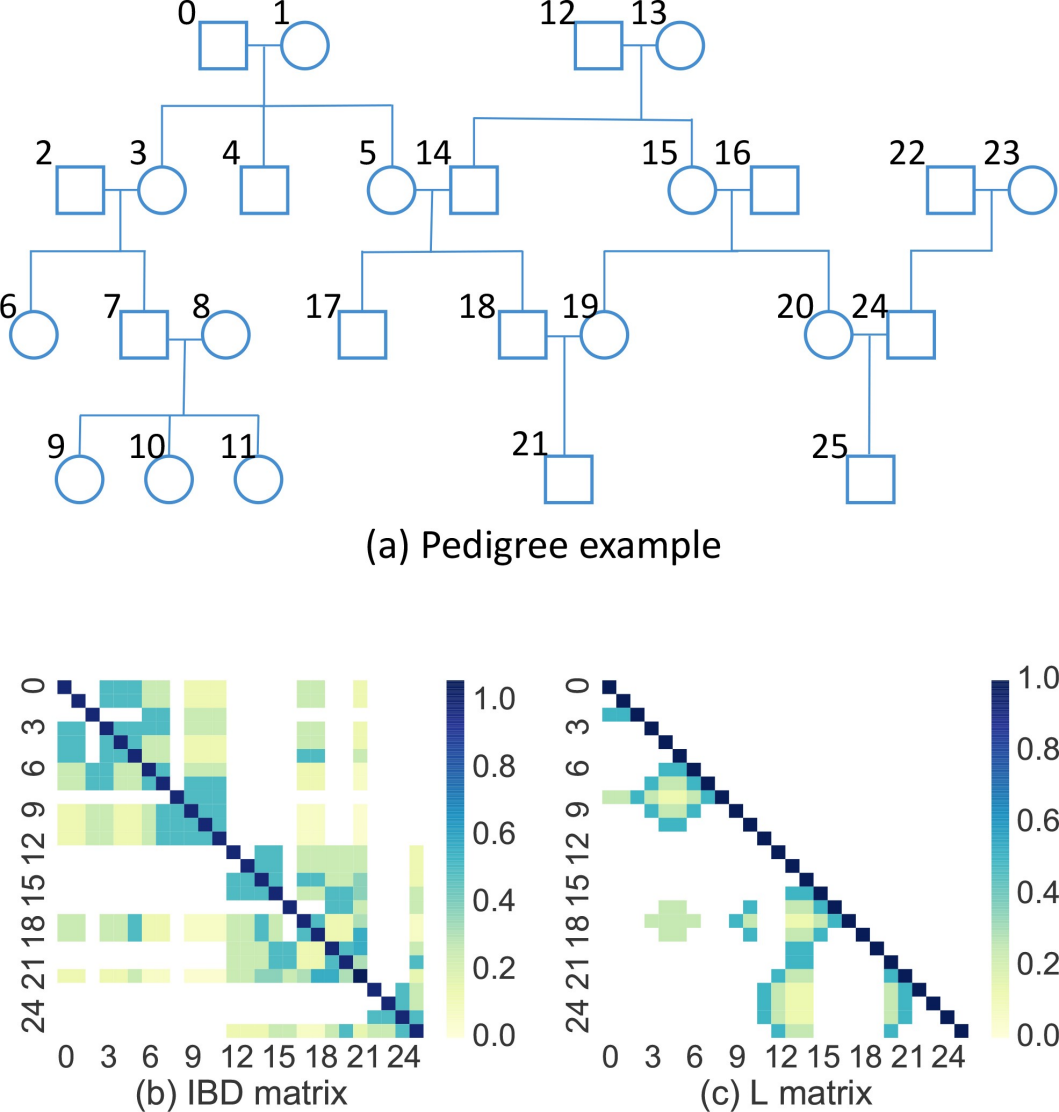
A demonstration of the Sci-LMM IBD matrix construction algorithm. (**a**) An example pedigree with 26 individuals. (**b**) A heat-map representing the IBD matrix, where zero elements are white to emphasize sparsity. (**c**) A heat-map representing the lower Cholesky factorization of the IBD matrix (i.e. the matrix *L* in the factorization ***A*** = ***LHL***^*T*^, where ***A*** is the IBD matrix). The value of entry *i*,*j* is the expected fraction of the genome that is shared between individual *i* and her ancestor *j*.

To efficiently compute the IBD matrix we first construct the matrices ***L*** and ***H*** of its decomposition ***A*** = ***LHL***^***T***^, where ***L*** is a lower triangular matrix such that ***L***_***ij***_ contains the fraction of genome shared between individuals *i* and her ancestor *j*, ***H*** is diagonal, and the matrices are ordered such that ancestors precede their descendants (Fig 1A–1C). The matrices ***L*** and ***H*** can be computed efficiently via iterative techniques [78,79] using sparse matrix routines [80] (S1 Text).

### Dominance kinship matrix

Dominancy represents the genetic variance due to co-ancestry of two alleles, and can be approximated by $\frac{1}{4}(A_{f_{i},f_{j}}\cdot A_{m_{i},m_{j}}+A_{f_{i},m_{j}}\cdot A_{m_{i},f_{j}})$, where *A*_*k*,*l*_ is the IBD coefficient of individuals *k*,*l*, and *f*_*k*_,*m*_*k*_ are the parents of individual k [10,87]. A necessary condition for nonzero dominancy entry is a nonzero IBD relationship, which enables rapid computation of the dominance matrix.

### Epistatic kinship matrix

Epistatic covariance encodes the assumption that variants interact multiplicatively to affect a given phenotype, and is proportional to the exponent of the corresponding IBD coefficient, i.e., (*A*_*k*,*l*_)^2^ for two-loci epistasis, (*A*_*k*,*l*_)^3^ for three-loci epistasis and so on [75]. Therefore, an epistatic covariance matrix is simple to compute given the IBD matrix.

### Pruning of uninformative individuals

Population scale pedigree data typically presents heterogeneity of the completeness of records. However, individuals with missing data may still be required for IBD computation. For example, consider a pedigree of two siblings with phenotypic data, and two parents and one uncle without phenotypic data. The parents are important for the IBD computation of the siblings, but the uncle is non-informative.

Sci-LMM applies pedigree-pruning techniques to remove non-informative individuals, similarly to other REML packages for pedigree analysis [70–73]. Briefly, we defined required individuals as individuals who have phenotypic and explanatory variables data, or individuals who appear in a lineage path connecting two individuals with such data with one of their least common ancestors (S1 Text; S1 Fig). This algorithm reduces the matrix construction time by several hours.

### Computing IBD principal components

In addition to covariance matrices, Sci-LMM can include the top principal components (PCs) of the IBD matrix as fixed effects, using sparse matrix routines [88]. The inclusion of PCs can capture major linear sources of variation in a dataset, and is motivated by large scale human genetic studies, where such PCs often capture population structure [89]. However, we caution that PCs computed from an IBD matrix are not guaranteed to capture population structure [90]. An alternative approach often employed in animal studies is the assignment of unobserved parents to genetic groups [91], but this approach requires knowledge about the location of birth of all individuals without known parents.

### Data simulations

We generated pedigrees mimicking real family patterns in the United States, partially based on publications by the United States Census Bureau [92,93]. We iteratively generated generations of individuals, where the first generation included two individuals, and the number of individuals in each successive generation increases by 40% (approximately the same ratio as in the GENI dataset), until obtaining the desired sample size. Each generation included 50% females and 50% males.

In each generation we generated households, where every household includes either one individual or two individuals with different genders, and every individual can belong to zero, one or multiple households. The number of households in each generation was 62.5% of the number of individuals in that generation. 68% of the households included pairs of individuals, and the rest included a single individual. Every individual in every generation (except for the top one) was born to parents from a randomly selected household from the previous generation (for 80% of individuals) or from two generations in the past (for the remaining 20% of individuals).

After generating all individuals, we omitted randomly selected edges until obtaining the desired sparsity factor, up to 10% error. We then created corresponding IBD, dominance and epistasis matrices.

Finally, we generated phenotypes using Eq 1 by (1) generating variance components $\sigma_{k}^{2}$ for each covariance matrix *M*^*k*^ from *U*(0,1) and scaling them such that they sum to 1.0; (2) Generating 5 binary and 5 normally distributed covariates; and (3) generating fixed effects from N(0,1000/n), where *n* is the sample size.

The parameters differentiating the various experiments are: (1) cohort size (50K, 100K, 250K, 500K, 1M or 2M); (2) sparsity factor (0.0005, 0.001, or 0.005); and (3) the subset of matrices used. We generated 10 different datasets for every unique combination of settings, except for matrices with 2M individuals, for which we generated a single pedigree with ten different phenotype vectors due to runtime considerations.

### Computing environment

All experiments were conducted using a Linux workstation with a 24-cores 2GHz Xeon E5 processor and 256Gb of RAM.

## Results

### Simulation studies

To evaluate the capabilities of Sci-LMM, we generated large synthetic pedigrees spanning 20 to 40 generations and various family structures, under a wide variety of settings. The pedigrees included 50,000–2,000,000 individuals, amounting to trillions of pairs of relatives. A subset of the individuals in each generation consists of children of individuals from either the previous generation, or from two generations in the past. To simulate patterns observed in real datasets, the simulations also included consanguinity, half-siblings, and individuals with less than two recorded parents (Methods).

In each simulation we generated a normally distributed phenotype, using a covariance matrix with additive, epistatic and dominance effects and ten binary covariates. Unless otherwise stated, the sparsity factor (the fraction of non-zero entries in each matrix) was 0.001. Ten different datasets were generated for each combination of sample size and sparsity factor.

**Fig 2:**
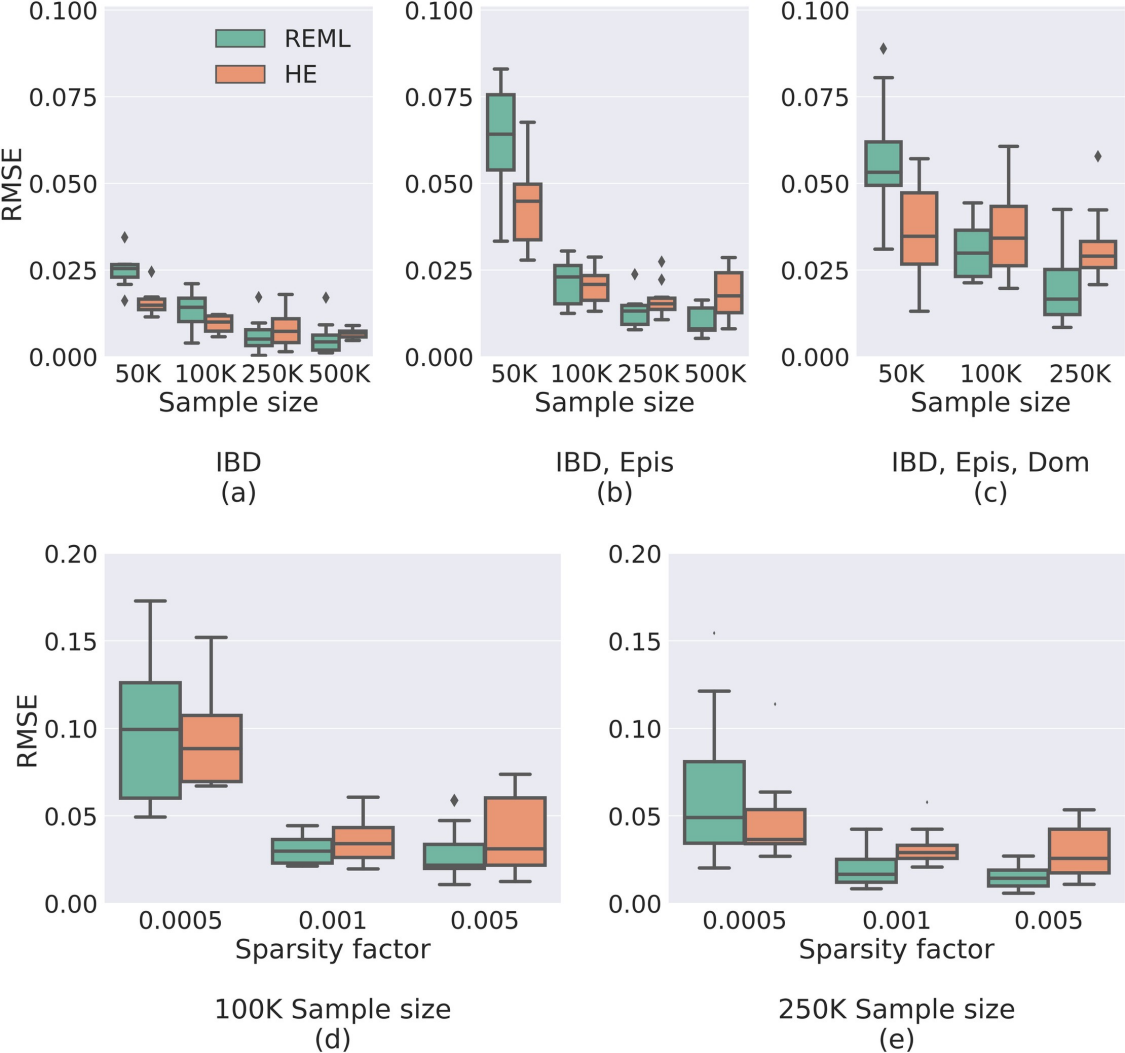
Evaluating the estimation accuracy of Sci-LMM. (**a-c**) Box plots comparing REML and HE estimation accuracy (RMSE) across simulated datasets (each box represents 10 experiments), under varying sample sizes, using **(a)** only IBD, **(b)** IBD and epistasis, or **(c)** IBD, epistasis and dominance variance components. HE is more accurate than REML for smaller sample sizes, but REML outperforms HE as the sample size increases. Results for analyses with three matrices and 500,000 individuals are omitted due to excessive required computational time. (**d-e**) Comparing REML and HE estimation accuracy when using IBD, epistasis and dominance matrices under various sparsity factors (the fraction of non-zero matrix entries) with either **(d)** 100,000 individuals, or (**e**) 250,000 individuals. The estimation accuracy of both REML and HE increases with the number of non-zero entries, for both REML and HE.

In all settings, Sci-LMM yielded empirically unbiased estimates of the variance components, using both REML and HE regression. As expected, estimation accuracy increased with sample size, though the estimators became slightly less accurate when increasing the number of variance components, (Fig 2A–2C). Specifically, the root mean square error (RMSE) was < 0.03 for all methods under all settings with more than 250,000 individuals, indicating <3% average error (because the phenotype was standardized to have unit variance).

A comparison of the REML and the HE results shows that that HE was slightly more accurate in the presence of <100,000 individuals (Fig 2A–2C), and REML was slightly more accurate otherwise. These results possibly indicate that REML convergence is difficult in the presence of sparse covariance matrices with limited sample sizes. We also found that estimation accuracy was anti-correlated with relatedness sparsity, indicating that the estimators efficiently exploit the information found in non-zero covariance entries (Fig 2D and 2E).

#### Runtime

**Fig 3:**
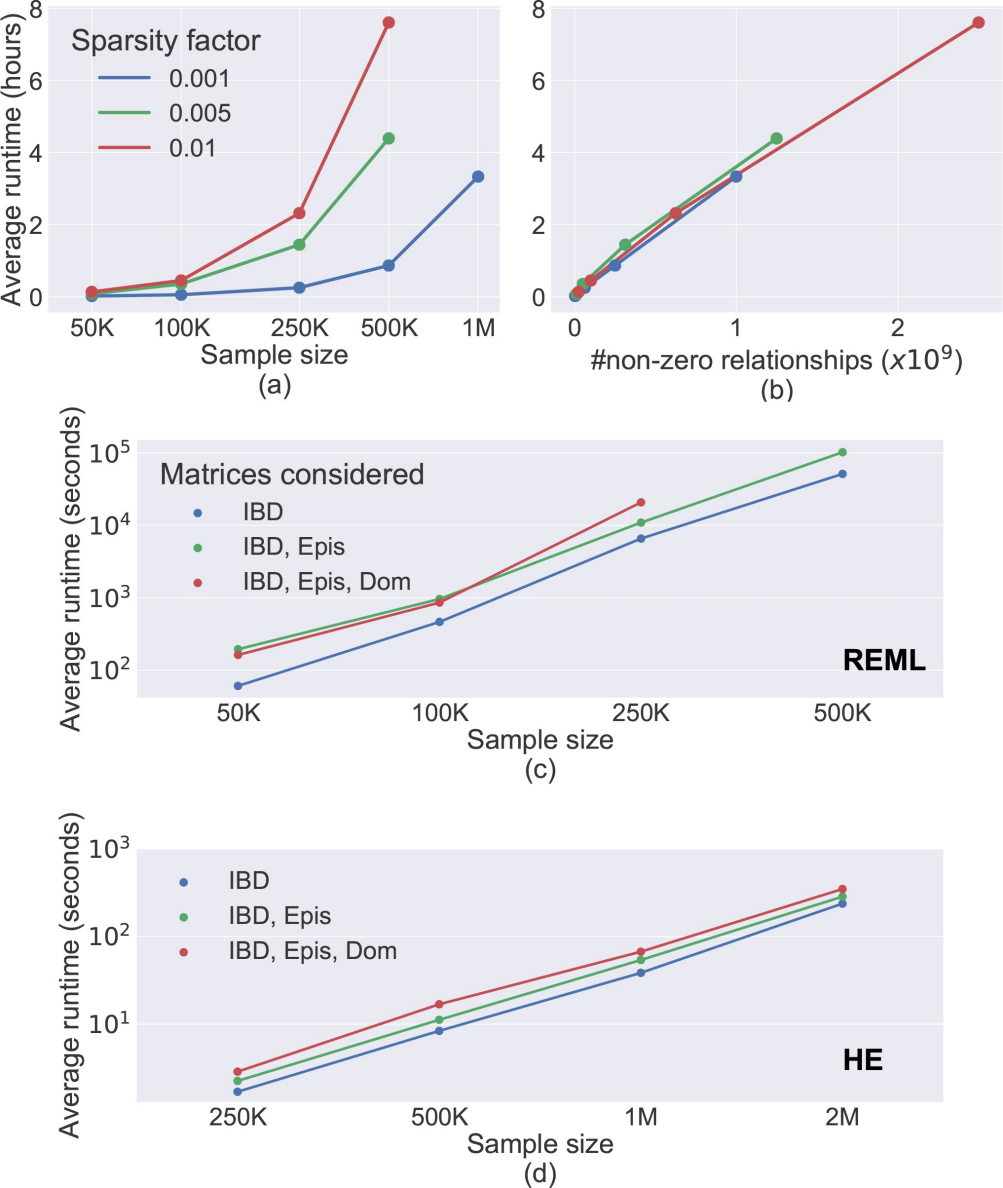
Analysis of Sci-LMM computation time. **(a)** Computation time required to compute an IBD matrix from pedigree data under different sparsity factors as a function of sample size. **(b)** Computation time required to compute an IBD matrix from pedigree data as a function of the number of nonzero relationships, demonstrating a linear relationship. The maximal number of evaluated non-zero relationships increases with the sparsity cutoff, because we only generated matrices with up to a million individuals. **(c)** Variance component estimation time (using REML), as a function of sample size, when using different combinations of covariance matrices. Epis–Epistasis; Dom—dominance **(d)** same as (c), but for HE regression instead of REML estimation. Here we evaluated datasets with up to 2 million individuals that were not investigated in (c), owing to technical limitations of the sparse matrix factorization routines used in our REML implementation.

We evaluated the time Sci-LMM requires to construct covariance matrices. The covariance matrices computation is dominated by the IBD matrix construction, because the other matrices can be computed trivially given this matrix (Methods). The IBD matrix construction scaled linearly with the number of non-zero entries in the matrix (Fig 3A and 3B). For example, Sci-LMM required less than 4 hours to construct an IBD matrix with 5×10^11^ pairs of possible relatives and a sparsity factor of 0.001.

We next investigated the runtime for variance component estimation using REML and HE. REML estimation for samples with 500,000 individuals (representing 250 million covariance entries) required less than 24 hours (Fig 3C), whereas HE estimation required 16 seconds (Fig 3D). Overall, our results demonstrate that the Sci-LMM framework is scalable to extremely large pedigrees.

We also compared Sci-LMM with several REML software packages [69–72]. We could not invoke any of these packages with an epistatic covariance matrix because they require its inverse, whose computation is more computational demanding than that of the IBD matrix [76]. We verified this by trying to invert the matrix analytically via the software package nadiv [94] and numerically via sparse matrix libraries [80] and via the matrix inversion facilities of the software package WOMBAT [71], all of which ran out of memory on a 256GB machine. We additionally tried running the analysis via the *fitNullModel* function of the GENESIS package [95] and the *lmekin* function of the coxme package [96], both of which can work with sparse covariance matrices. However, both packages could not complete the analysis in 4 days, presumably because they do not use the approximate gradient approximation techniques of Sci-LMM.

Finally, we compared Sci-LMM to WOMBAT in the presence of only an additive covariance matrix. WOMBAT is more computationally efficient in this setting because it uses a mixed model equations (MME) solver [47]. MME solvers scale roughly quadratically with the sample size, compared with the cubic complexity of Sci-LMM, but they require pre-computing the inverse of the LMM covariance matrix. Thus, using an MME solver is advantageous in the presence of only an IBD covariance matrix, whose inverse has an analytical form that can be computed efficiently [78,79]. Indeed, WOMBAT was much faster than the REML solver of Sci-LMM in this setting, completing an analysis of 250,000 individuals in 13 minutes, compared with 164 minutes for Sci-LMM (S1 Table). However, both WOMBAT and the REML solver of Sci-LMM crashed in the presence of ≥500,000 individuals, whereas the HE solver of Sci-LMM could complete the analysis in less than 20 minutes. Hence, the HE solver of Sci-LMM is the only tool that we are aware of that can readily scale to population scale pedigrees.

#### Estimating the heritability of longevity and reproductive fitness

We used Sci-LMM to estimate the heritability of longevity and reproductive fitness, based on large-scale pedigree records obtained from the Geni genealogical website [1] (see Web Resources). An initial description of the longevity analysis was reported in [1], but here we substantially refine and extend this analysis. We applied stringent quality control to minimize deaths due to non-natural reasons such as wars or natural disasters, by excluding pairs of individuals who died within 10 days of each other or within periods with significantly elevated death rates [1]. This filtering yielded approximately 441,000 individuals with birth and death dates. We first computed the IBD, dominance and epistasis matrices of these individuals, and then estimated the heritability of longevity using these matrices.

**Fig 4:**
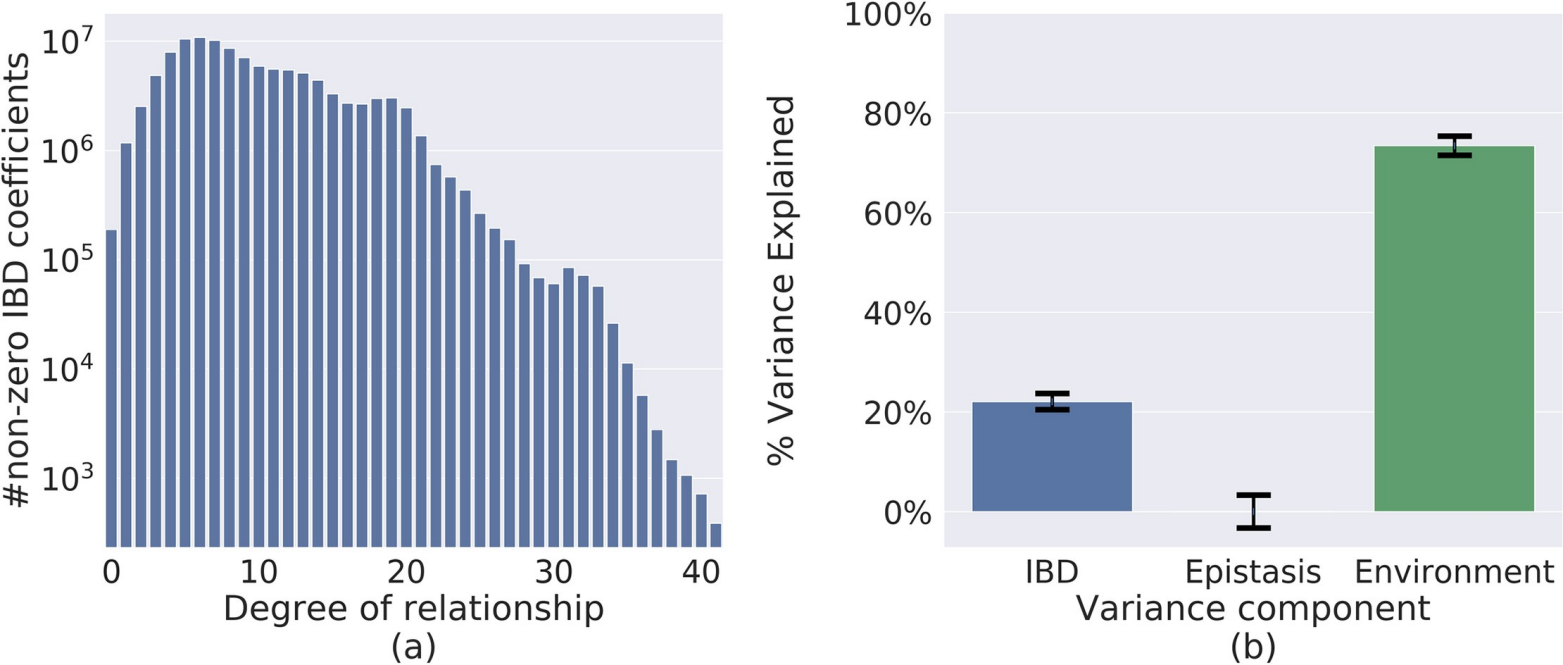
Results of analysis of a real pedigree with 441,000 individuals. **(a)** A histogram of genetic similarity across 441,000 individuals, using only the closest relationship between every pair of individuals. The degree of relationship between a pair of individuals is given by −log_2_(*K*_*ij*_)−1, where *K*_*ij*_ is their IBD coefficient (Methods). The dataset includes approximately 9.7 million pairs of individuals whose least common ancestor lived at least 10 generations earlier. **(b)** The estimated fraction of longevity variance attributed to different variance components (and their 95% CI).

The corresponding IBD matrix contained over 3 billion nonzero entries. It included the 441,000 core individuals and their informative ancestors, yielding a total of 1.6 million individuals. The submatrix consisting of only the core individuals included 251 million non-zero IBD coefficients (yielding a sparsity factor of ~0.001, in correspondence with the simulation studies). The dataset included 9.7 million pairs of individuals with a kinship coefficient corresponding to a > = 20-degree relationship (Fig 4A). Sci-LMM constructed this matrix in 10 hours.

Next, we used Sci-LMM to estimate the heritability of longevity with covariates encoding gender, year of birth (yob), yob raised to second and third power, and the top 10 principal components of the IBD matrix, and with covariance matrices encoding IBD and pairwise epistasis (Methods). A dominance matrix was not included because the analysis included a relatively small number of full-sibs or double first cousins, rendering this matrix almost equivalent to the identity matrix (S2 Fig).

The Sci-LMM REML estimates were: IBD: 22.1% (s.e. 0.8%); pair-wise epistasis: 0.001% (s.e. 1.7%); environmental effects: 74.4% (s.e. 1.0%) (Fig 4B). The HE estimate for IBD was 24.3% (s.e. 0.5%) when omitting the epistatic interactions matrix (HE results with epistatic interactions were inconclusive due to large standard errors). A potential challenge of our framework is that genetic covariance may be correlated with shared environmental factors (Methods) [2,97]. We tried mitigating this problem by excluding ~399,000 pairs (~0.15%) of individuals with a shared household (spouses or parent-child pairs) from the analysis without excluding the individuals themselves, using HE (Methods). This led to a heritability estimate of 26.3% (s.e. 0.9%), indicating that shared household effects are unlikely to up-bias our estimates. Overall, our results suggest that the heritability of longevity is upper bounded by ~26%. However, the true heritability may be lower since our estimates may be confounded by other environmental factors [2,97] (see Discussion).

We next estimated the heritability of reproductive fitness, quantified by number of children. To limit confounding due to non-genetic factors, we applied stringent filtering of individuals. We removed individuals with less than two children records, because the family records of such individuals are more likely to be incomplete. We additionally removed individuals with a shared household (spouses and children) and individuals who are first- or second-degree relatives of another individual in the data set. The filtered dataset included ~45,000 individuals. We used the same covariates as before, applied a Box-cox transformation to induce normality for number of children, and excluded epistatic interactions from this reduced dataset, because they led to large standard errors.

The REML and HE estimated heritability of reproductive fitness were 28.4% (s.e. 0.5%) and 34.4% (s.e. 1.2%), respectively. These results indicate a substantial genetic component for reproductive fitness, in line with previous work [98]. However, we note several potential caveats. First, our analysis estimated the heritability of reproductive fitness conditional on having ≥2 children; we excluded individuals with fewer children to minimize bias towards individuals with more complete family records. Second, our estimate may be upper-biased due to confounding by non-genetic factors [2,97] (see Discussion). Third, our analysis may be improved by including random effect for shared households or other shared environmental factors. However, such analyses require the introduction of additional modeling assumptions, which we leave for future work.

Finally, we performed a series of experiments to examine the robustness of our longevity analysis to potential confounding factors (S2 Table). First, we restricted the analysis to 283,073 individuals born after 1800, which yielded heritability estimates of 0.23 (0.006) under HE and 0.23 (0.004) under REML. Second, we restricted the analysis to 276,011 individuals who are unlikely to have shared a household during their lifetime (i.e., no spouses or first-degree relatives), which yielded estimates of 0.29 (0.006) under HE and 0.29 (0.005) under REML. Third, we restricted the analysis to 110,237 individuals who were not first or second-degree relatives, which led to estimates of 0.53 (0.040) under HE and 0.51 (0.040) under REML. Finally, we combined the last two restrictions by only including 106,049 individuals who were neither spouses or first or second-degree relatives of each other, leading to estimates of 0.54 (0.045) under HE and 0.51 (0.044) under REML. The inflated estimates when excluding first or second-degree relatives may indicate that weaker levels of IBD covariance are correlated with shared environmental covariance, leading to inflated heritability estimates. Nevertheless, these results indicate that our original analysis is not upper biased due to inclusion of the above potential confounding factors.

## Discussion

We have described a statistical framework for analysis of large pedigree records spanning millions of individuals. Our framework includes methodologies for constructing large sparse matrices given raw pedigree data, and methodologies for LMM analysis with random effects described by these matrices. Taken together, the proposed solution enables an end-to-end analysis of population scale human family trees.

In this work we focused on partitioning phenotypic variance into additive genetics, epistasis and dominance. However, the LMM framework is flexible and can be extended in various directions. For example, sparse LMMs are often used to model transmissible phenomena [99–104], which enables combining pedigree-based and geography-based covariance structures. Both Sci-LMM and the data studied in this paper are freely available for download, which makes the analysis of population-scale human family trees widely accessible to the research community. Combined, these resources allow researchers to investigate genetic and epidemiological questions on unprecedented scales.

We evaluated two methods for variance components estimation: REML and HE regression. REML is more accurate than HE and provides a likelihood-based solution, which can be used for model comparison and hypothesis testing. HE estimates are less accurate but can be more robust to modeling violation. Importantly, HE regression can mitigate confounding due to environmental factors by zeroing selected entries in the covariance matrices, which may be especially suitable for studying human genealogical records (Methods). Hence, the two methods are complementary in terms of their strengths and weaknesses. In practice, we found that it is difficult to scale REML to datasets with more than 500,000 individuals with a sparsity factor of 0.001. Our recommendation is to use REML when it is feasible and all model assumptions hold, and HE regression otherwise. We note that REML estimation can be substantially faster when not fitting epistatic interactions by using a mixed model equations approach [47], which is implemented in several software packages [69–72].

Our work demonstrates the technical feasibility of studying population scale human family trees. However, the analysis of human genealogical records is challenging due to imperfect data and the difficulty of controlling for confounding factors. Potential issues include non-paternity, cryptic relatedness, missing or false genealogical records, genetic nurture [105,106], environmental bias [97,107], assortative mating [2] and correlation between additive and epistatic effects [17,18]. As such, our estimates should be considered as a first order approximation, and our heritability estimates are likely upper biased due to confounding. We expect that recently proposed techniques to address these issues (e.g. [2,106,107]) could be integrated into the Sci-LMM framework in the future.

In this work we focused on analyzing large pedigree records with no measured genotypes. In recent years, the advent of biobank-sized datasets allows analyzing population-scale genotyped cohorts. The two study types are complementary because biobanks cannot be used to investigate longevity, traits with a late age of onset, or epidemiological and sociological questions on historical scales. We anticipate that cohorts combining both types of data will become increasingly common. For example, we and other online genealogy platforms allow users to upload their genetic information and link it with their genealogical profile. Such combined datasets have been extensively explored in the animal breeding literature [19,21,108–112]. However, privacy and logistical concerns limit public access to human genetic data, necessitating methods based on summary statistics [61]. Thus, approaches for analysis of such combined datasets will combine state of the art techniques from the animal breeding and the human genetics literature, and remain a potential avenue for future work.

## Supporting information

S1 Text Detailed algorithms for covariance matrix construction and pedigree pruning. (PDF)

S1 Table Comparison of Sci-LMM and WOMBAT runtime and memory requirements, when using simulations with only an additive IBD matrix. Both tools used only 1 CPU thread, and WOMBAT was executed with the—meuwissen flag. The estimated values were essentially the same for both tools in all cases. WOMBAT and Sci-LMM REML both crashed in the presence of pedigrees with ≥500,000 individuals.
(PDF)

S2 Table Sci-LMM heritability of longevity estimates under different analysis approaches. The epistasis estimates were very close to zero in all cases and are omitted for clarity.
(PDF)

S1 Fig Stages of removal of uninformative individuals. Nodes represent individuals, and edges represent parent-child relations. Only red individuals have full information records (e.g. year of birth, year of death, etc.)
(TIF)

S2 Fig The number of nonzero IBD and Dominance entries in the GENI dataset, as a function of degree of relationship. (TIF)
