# Supplementary material for: Estimating variance components in population scale family trees
Source: PLoS Genet. 2019 May 9;15(5):e1008124. doi: 10.1371/journal.pgen.1008124 (PMC6529016; doi:10.1371/journal.pgen.1008124)
Supplement: S1 Text — (PDF) [file pgen.1008124.s001.pdf]

Estimating variance components in population scale family trees: Supplementary Text S1

1 Constructing the IBD matrix

The algorithm for constructing the IBD matrix is presented below.

Algorithm 1 Computing an IBD matrix

Definitions

- $CSRM(n, m)$ - Compressed Sparse Row $\mathbb{R}^{n\times m}$ matrix [3]
- $RLL\_SM(n, m)$ - Row-based linked list sparse $\mathbb{R}^{n\times m}$ matrix

Parameters

- $ParentsList$ - individuals' parents

Algorithm

$\boldsymbol{L = RLL\_SM(N, N)}$
$H,\ F = \bar{0}_N$
for $i = 0;\ \ i < n$ **do**
    $L[i, i] = 1;\ \ ANC = i;\ \ iParents = ParentsList[i]$
    $H[i] = 1 - 0.25 \cdot (count(iParents) + sum(F[iParents]))$
    while $count(ANC) > 0$ **do**
        $j = max(ANC);\ jParents = AncestorList[j]$
        $L[i, jParents] \mathrel{+}= 0.5 \cdot L[i, j]$
        $F[i] \mathrel{+}= L[i, j]^2 \cdot H[i]$
        $ANC = ANC\ \cup jParents \setminus \{j\}$
    end while
    $F[i] \mathrel{-}= 1$
end for
$L\_CSRM = CSRM(L)$
$H\_CSRM = CSRM(withdiagonalH)$
return $L\_CSRM \times H\_CSRM \times L\_CSRM^T$

2 Removing non-informative individuals

The Familinx dataset includes $N_0 = 43M$ individuals. Of these, only $N = 441K$ were selected by Kaplanis *et al.* as eligible individuals (i.e., individuals who passed various filtering criteria, such as not likely to have died due to non-natural causes, who have records of year of birth and year of death, etc.). However, a subset of the non-eligible individuals is still required for IBD computation purposes. For example, an individual with no year of birth, that is a parent of two eligible individuals, is still required for encoding the information that these two individuals are siblings.

We therefore distinguish between *eligible* individuals, which are the individuals selected by Kaplanis *et al.* and *informative individuals*, who are either (1) eligible, or (2) non-eligible but are required for IBD computation purposes.

The first stage of our IBD computation procedure consists of removing uninformative individuals. By using the definition of IBD (defined in the main text), the list of informative individuals consists of (1) eligible individuals; and (2) individuals who appear in a path connecting two eligible individuals with their least common ancestors.

We performed the pruning in three steps. First, we sorted individuals such that every individual precedes her offspring, using the networkx package [2].

Second, we removed non-eligible individuals who are not ancestors of any eligible individual. To perform this pruning efficiently, we created a matrix AM such that (1) $AM_{ij} > 0$ only if there is a path of parent-child links connecting individuals i and j; and (2) $i > j$ only if individual j is not an offspring of individual i. By denoting rel as a binary adjacency matrix of parent-child pairs with the same ordering, we can compute the desired matrix AM as follows:

$$AM = \sum_{i=1}^{oa} rel^i, \tag{1}$$

where oa represents the longest path between an individual and her ancestor. This expression exploits the fact that for an adjacency matrix M, the matrix M^d (for some integer $d > 0$) is a matrix of distances, such that $M_{i,j}$ is the number of paths of length d between individuals i and j. The AM matrix can be efficiently computed with compressed row matrices [1].

Given the matrix AM, we can trivially remove all non-eligible individuals that have no eligible offspring.

In the next step, we removed non-eligible individuals who are not in any path between two eligible individuals passing through a common ancestor. For this, we computed the matrix $CAM = (AM + I) \times (AM + I)^T$ and used the results of Lemma 1 to efficiently find all such individuals.

Lemma 1. *For every pair of individuals i and j, $CAM[i, j] > 0$ if and only if i and j share common ancestors, where CAM is defined as $(AM + I) \times (AM + I)^T$*

Proof. We first write down the explicit form of an arbitrary entry $CAM[i, j]$:

$$\begin{aligned} CAM[i,j] &= \sum_k (AM+I)[i,k] \cdot (AM+I)[j,k] \\ &= \sum_k AM[i,k]AM[j,k] + AM[i,k]I[j,k] + AM[j,k]I[i,k] + I[i,k]I[j,k] \\ &= \sum_k AM[i,k]AM[j,k] + \sum_k AM[i,k]I[j,k] + \sum_k AM[j,k]I[i,k] + \sum_k I[i,k]I[j,k] \end{aligned}$$

Since AM is non-negative, we conclude that $\boldsymbol{CAM}[i, j] > 0$ if and only there exists at least one index k such that at least one of the following conditions hold:

1. $\boldsymbol{AM}[i, k]\boldsymbol{AM}[j, k] > 0$
2. $\boldsymbol{AM}[i, k]\boldsymbol{I}[j, k] > 0$

3. $\boldsymbol{AM}[j,k]\boldsymbol{I}[i,k] > 0$

4. $\boldsymbol{I}[i,k]\boldsymbol{I}[j,k] > 0$

If condition 1 holds, individual k is a common ancestor of individuals i and j. If condition 2 holds, $j = k$ and individual j is an ancestor of individual i. If condition 3 holds, the converse statement holds. Finally, if condition 4 holds, $i = j = k$, and so the individuals trivially share an ancestor.

■ □

References

[1] A. Buluç, J. T. Fineman, M. Frigo, J. R. Gilbert, and Leiserson C. E. Parallel Sparse Matrix-Vector and Matrix-Transpose-Vector Multiplication Using Compressed Sparse Blocks. *SPAA*, 2009.

[2] Aric A. Hagberg, Daniel A. Schult, and Pieter J. Swart. Exploring network structure, dynamics, and function using NetworkX. In *Proceedings of the 7th Python in Science Conference (SciPy2008)*, pages 11–15, Pasadena, CA USA, August 2008.

[3] Danny C Sorensen. Implicitly restarted Arnoldi/Lanczos methods for large scale eigenvalue calculations. In *Parallel Numerical Algorithms*, pages 119–165. Springer, 1997.
