# Supplementary material for: Estimating variance components in population scale family trees
Source: PLoS Genet. 2019 May 9;15(5):e1008124. doi: 10.1371/journal.pgen.1008124 (PMC6529016; doi:10.1371/journal.pgen.1008124)
Supplement: S1 Table — Both tools used only 1 CPU thread, and WOMBAT was executed with the—meuwissen flag. The estimated values were essentially the same for both tools in all cases. WOMBAT and Sci-LMM REML both crashed in the presence of pedigrees with ≥500,000 individuals. (PDF) [file pgen.1008124.s002.pdf]

Supplementary Table 1

Sample size	Method	Time (minutes)	Peak memory (Megabytes)
10000	Sci-LMM (IBD computation)	0.23	111
	Sci-LMM (HE)	0.02	68
	Sci-LMM (REML)	0.02	101
	WOMBAT	0.03	232
100000	Sci-LMM (IBD computation)	4.0	306
	Sci-LMM (HE)	1.07	631
	Sci-LMM (REML)	2.3	1462
	WOMBAT	1.3	232
250000	Sci-LMM (IBD computation)	14.0	1032
	Sci-LMM (HE)	5.3	2201
	Sci-LMM (REML)	150.0	12443
	WOMBAT	12.7	471
500000	Sci-LMM (IBD computation)	27.0	3621
	Sci-LMM (HE)	12.75	8904
	Sci-LMM (REML)	-	-
	WOMBAT	-	-
