# Supplementary material for: Estimating variance components in population scale family trees
Source: PLoS Genet. 2019 May 9;15(5):e1008124. doi: 10.1371/journal.pgen.1008124 (PMC6529016; doi:10.1371/journal.pgen.1008124)
Supplement: S2 Table — The epistasis estimates were very close to zero in all cases and are omitted for clarity. (PDF) [file pgen.1008124.s003.pdf]

Supplementary Table 2

Individuals to exclude	sample size	IBD (HE)	IBD (REML)
born before 1800	283073	0.23 (0.006)	0.23 (0.004)
in same household	276011	0.29 (0.006)	0.28 (0.005)
1st or 2nd degree relatives	110237	0.53 (0.040)	0.51 (0.040)
In same household or $\leq$ 2nd degree relatives	106469	0.539 (0.045)	0.51 (0.044)
