# Supplementary figures and images for: Estimating variance components in population scale family trees
Source: PLoS Genet. 2019 May 9;15(5):e1008124. doi: 10.1371/journal.pgen.1008124 (PMC6529016; doi:10.1371/journal.pgen.1008124)

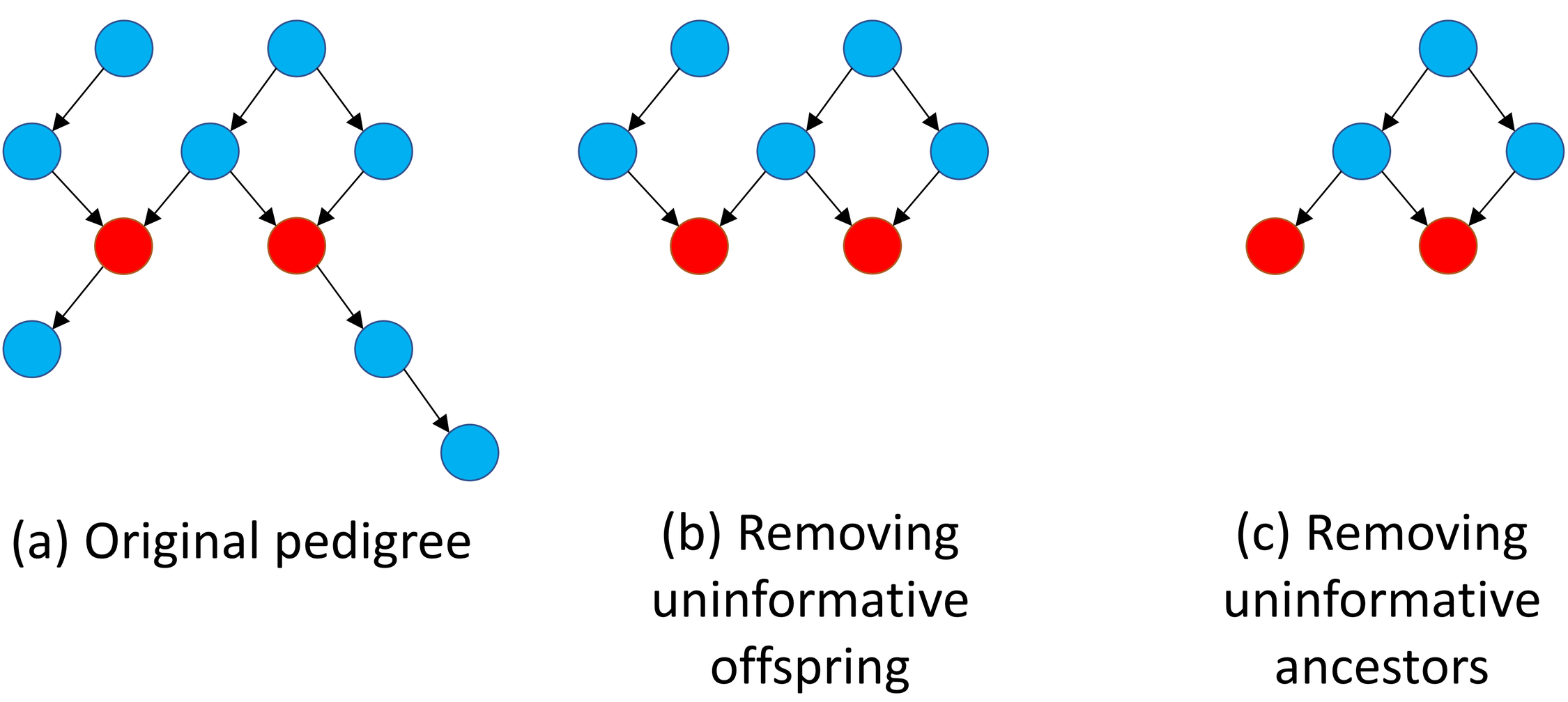

Supplement: S1 Fig — Nodes represent individuals, and edges represent parent-child relations. Only red individuals have full information records (e.g. year of birth, year of death, etc.) (TIF) [file pgen.1008124.s004.tif]

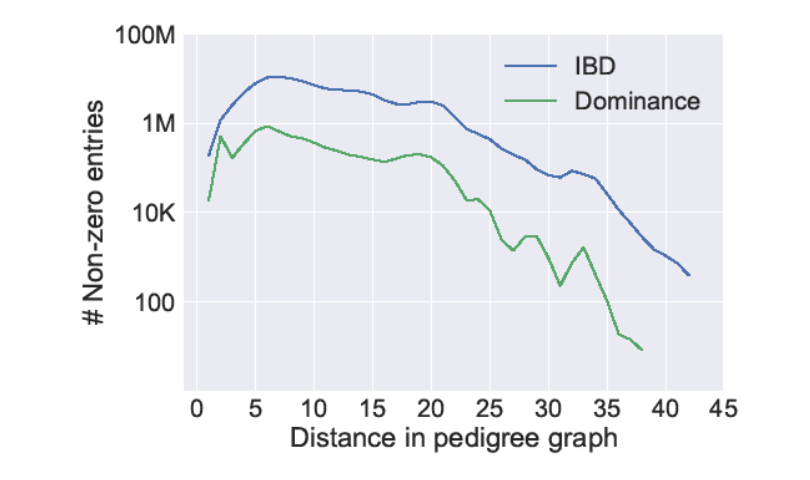

Supplement: S2 Fig — (TIF) [file pgen.1008124.s005.tif]
